# Supplementary material for: Extending on-surface synthesis from 2D to 3D by cycloaddition with C60
Source: Nat Commun. 2023 Sep 28;14:6075. doi: 10.1038/s41467-023-41913-3 (PMC10539376; doi:10.1038/s41467-023-41913-3)
Supplement: Supplementary file 1 — Supplementary Information [file 41467_2023_41913_MOESM1_ESM.pdf]

Supplementary Information

Extending on-surface synthesis from 2D to 3D by cycloaddition with C_{60}

Pengcheng Ding[1], Shaoshan Wang[1], Cristina Mattioli[2], Zhuo Li[1], Guoqiang Shi[1], Ye Sun[3], André Gourdon[2], Lev Kantorovich[4], Flemming Besenbacher[5], Federico Rosei[6], and Miao Yu[1,*]

1. School of Chemistry and Chemical Engineering, Harbin Institute of Technology, Harbin 150001, China

2. CEMES-CNRS, Toulouse 31055, France.

3. School of Instrumentation Science and Engineering, Harbin Institute of Technology, Harbin 150001, China

4. Department of Physics, King's College London, The Strand, London WC2R 2LS, United Kingdom

5. Interdisciplinary Nanoscience Center (iNANO), Aarhus University, Aarhus 8000, Denmark

6. INRS Centre for Energy, Materials and Telecommunications, Varennes J3X 1P7, Canada

Correspondence to: miaoyu_che@hit.edu.cn

Supplementary Figures

1. Extended BCPM network on Au(111)

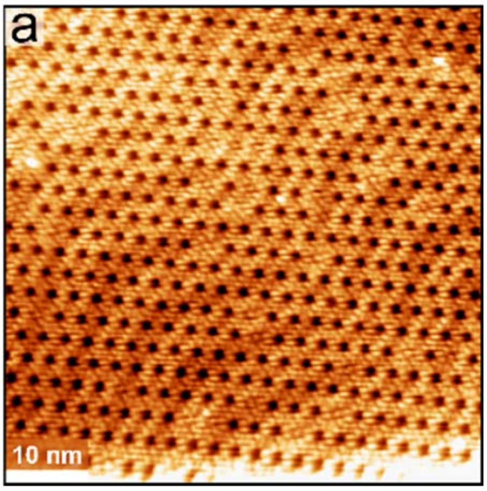

Supplementary Fig. 1 | Large-scale STM image of the extended porous network of BCPM on Au(111), showing the long-range order of the molecules.

2. C_{60} layer sitting on the BCPM layer

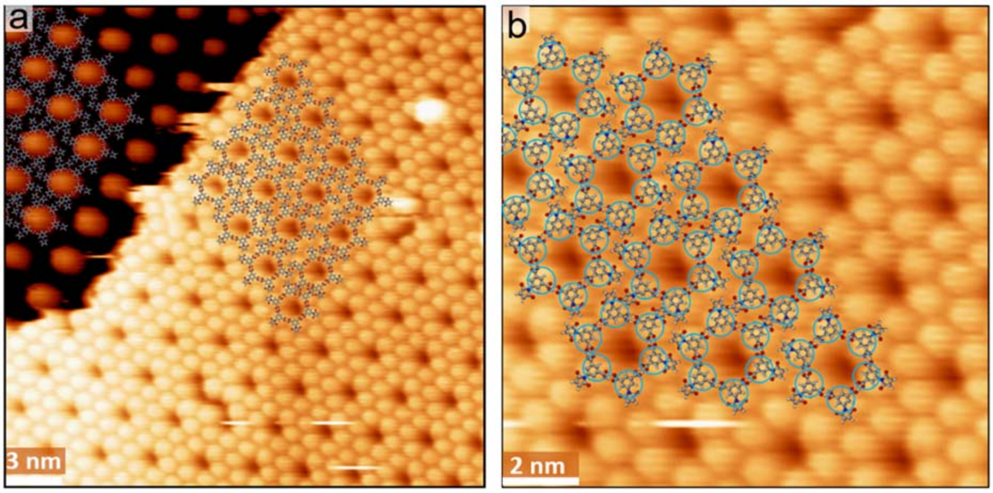

Supplementary Fig. 2 | a, Large-scale and **b,** close-view STM images of C_{60} layers on the BCPM-coated Au(111). In panel a, the structural model of BCPM network is superimposed, where the dark section corresponds to the BCPM layer with the C_{60} molecules hosted in the pores of the network and the bright zone corresponds to C_{60} molecules sitting on the BCPM layer. To indicate the relative position of C_{60} on the BCPM layer, the structural model of BCPM network is superimposed with the positions of C_{60} marked by the blue circles in panel b, showing that each C_{60} sits exactly on top of the phenyl ring of every BCPM molecule.

3. Analysis of the frontier orbitals for [4+2] cycloaddition between BCPM and C_{60}

Frontier molecular orbital theory was employed to understand [4+2] cycloaddition between BCPM and C_{60}, where the interactions between HOMO and LUMO of the reactants were considered [1]. As shown in Supplementary Fig. 3, the HOMO of BCPM is essentially located at the phenyl ring, and its lowest LUMO is localized around the maleimide ring. As a typical dienophile, C_{60} has a relatively low-lying LUMO and its [6,6] bonds can act as preferential sites for [4+2] cycloaddition [2–4]. Upon adsorption of C_{60} on the BCPM layer, after the pores of BCMP layer are occupied by the guest C_{60} molecules, each excess C_{60} molecule sits exactly on top of one BCPM's phenyl ring whilst the mobility of C_{60} moving to the top of the maleimide ring is constrained (Fig. 4c). The steric geometry between C_{60} and BCPM allows the overlap hence hybridization only between the HOMO of BCPM and the LUMO of C_{60} (Supplementary Figs. 3c and 3g). The energy difference between these molecular orbitals is 3.78 eV, which is suitable to form a bonding orbital for cycloaddition [5]. Moreover, the wave function of the LUMO of BCPM and HOMO of C_{60} also show a good match in the orbital symmetry for hybridization. As there is no overlap between the LUMO of BCPM and the HOMO of C_{60} due to this steric hindrance (Supplementary Figs. 3f), their inverse [4+2] cycloaddition cannot take place.

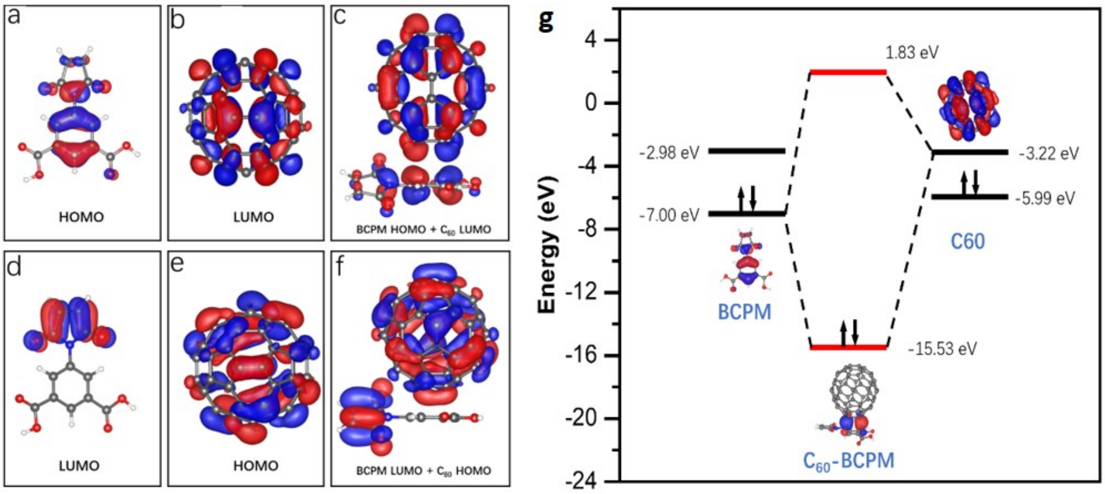

Supplementary Fig. 3 | a–c, Calculated HOMO of BCPM, LUMO of C_{60} and the hybridization between the HOMO of BCPM and the LUMO of C_{60} when C_{60} sitting on the phenyl ring of BCPM. **d–f,** Calculated LUMO of BCPM, HOMO of C_{60} and the non-overlapped LUMO of BCPM and HOMO of C_{60} in the given steric configuration. **g,** Energy level diagram of the LUMO and HOMO of BCPM and C_{60} together with the bonding orbital and anti-bonding orbital of their adduct, i.e., C_{60}-BCPM.

4. Varied intermolecular hydrogen bonding of BCPMs induced by their cycloaddition with C_{60}

As C_{60}-BCPM molecule consists of BCPM and C_{60} parts, the interactions between two C_{60}-BCPMs involve both hydrogen bonding (H-bonding) between the BCPMs and vdW interaction between the C_{60} parts. The hydrogen bonding energy is thus calculated by the following equation:

$$E_{\mathrm{H-bonding}} = E_{\mathrm{Total}} - 2 \times E_{\mathrm{Single\ C_{60}\text{-}BCPM}} - E_{\mathrm{vdW\ of\ C_{60}\ parts}}$$

Where E_{Total} is the total energy of the two C_{60}-BCPMs, $E_{\mathrm{Single\ C_{60}\text{-}BCPM}}$ is the energy of a single C_{60}-BCPM that is full relaxed in gas phase, and $E_{\mathrm{vdW\ of\ C_{60}\ parts}}$ is the interaction energy calculated with the C_{60} parts separated from the two C_{60}-BCPM molecules. The calculated H-bonding energy between two C_{60}-BCPMs is only 0.58 eV, much lower than that of two pristine BCPMs (1.2 eV). The reduced H-bonding is attributed to the nonplanar BCPM part hence tilted carboxyl groups induced by [4+2] cycloaddition of BCPM with C_{60}.

Since BCPMs self-assemble into the honeycomb network whilst C_{60} molecules prefer the close-packed arrangement, the stability of honeycomb network of C_{60}-BCPMs was compared with that of their close-packed arrangement. The stabilization energy per molecule is calculated using the following equation:

$$E_{\mathrm{Stablization}} = \frac{E_{\mathrm{Total}} - n \times E_{\mathrm{Single\ C_{60}\text{-}BCPM}}}{n}$$

where E_{Total} is the total energy of the molecular unit cell, *n* is the number of C_{60}-BCPMs in the cell, and $E_{\mathrm{Single\ C_{60}\text{-}BCPM}}$ is the energy of one single C_{60}-BCPM full relaxed in gas phase. After full relaxation of the atomic positions and lattice vectors, the stabilization energy of honeycomb network of C_{60}-BCPMs is calculated to be –1.60 eV; in contrast, the stabilization energy of the close-packed C_{60}-BCPMs is –2.78 eV.

The results reveal that cycloaddition weakens the intermolecular H-bonding between BCPMs and the close-packed arrangement of C_{60}-BCPMs is more stable, explaining the different packing structure of C_{60}-BCPM from that before cycloaddition.

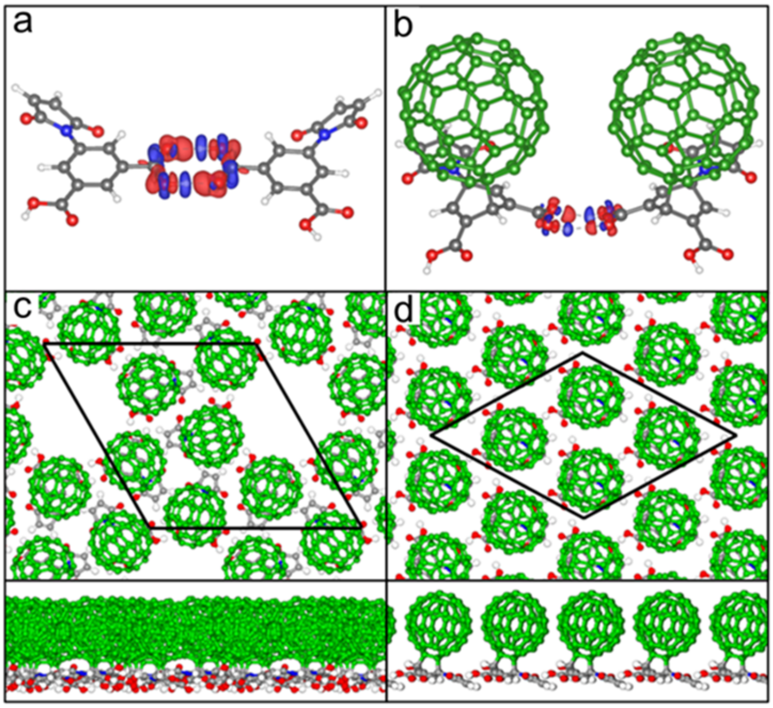

Supplementary Fig. 4 | Electron density difference plot showing H-bonding of **a,** two BCPMs, and **b,** two C_{60}-BCPMs, where the isosurface value is 0.02 e/Å^3. DFT-optimized structure of **c**, the honeycomb network (same to that of C_{60}-on-BCPM) and **d**, the close-packed arrangement of C_{60}-BCPMs.

5. Diffusion barrier of a single BCPM, C_{60} and C_{60}-BCPM on Au(111)

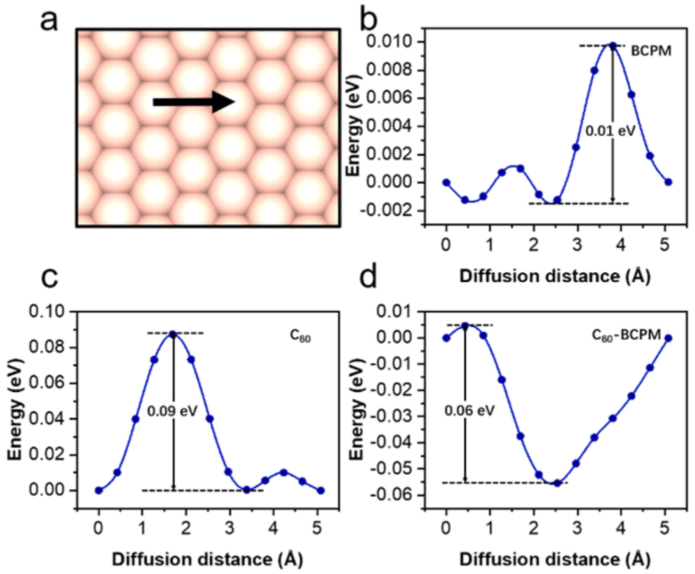

Supplementary Fig. 5| a, The model of Au(111) surface, where the black arrow shows the diffusion path of the molecule for the calculation of Minimum Energy Path (MEP) profiles. The calculated MEP profile for **b,** a single BCPM, **c,** a single C_{60} and **d,** a single C_{60}-BCPM on Au(111).

6. Macrocycles formed by annealing C_{60}-BCPM at 490 K for 30 min

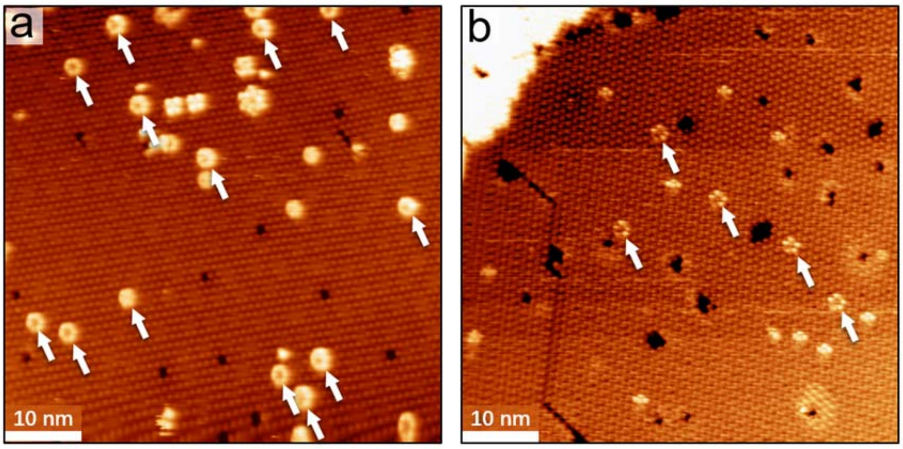

Supplementary Fig. 6 | a–b, STM images showing macrocycles formed by annealing C_{60}-BCPM at 490 K for 30 min, where hexamer ring (pointed out by the white arrows) is the primary form for the macrocycles.

7. Successive STM scanning for the hexamer ring with various tunneling parameters at room temperature

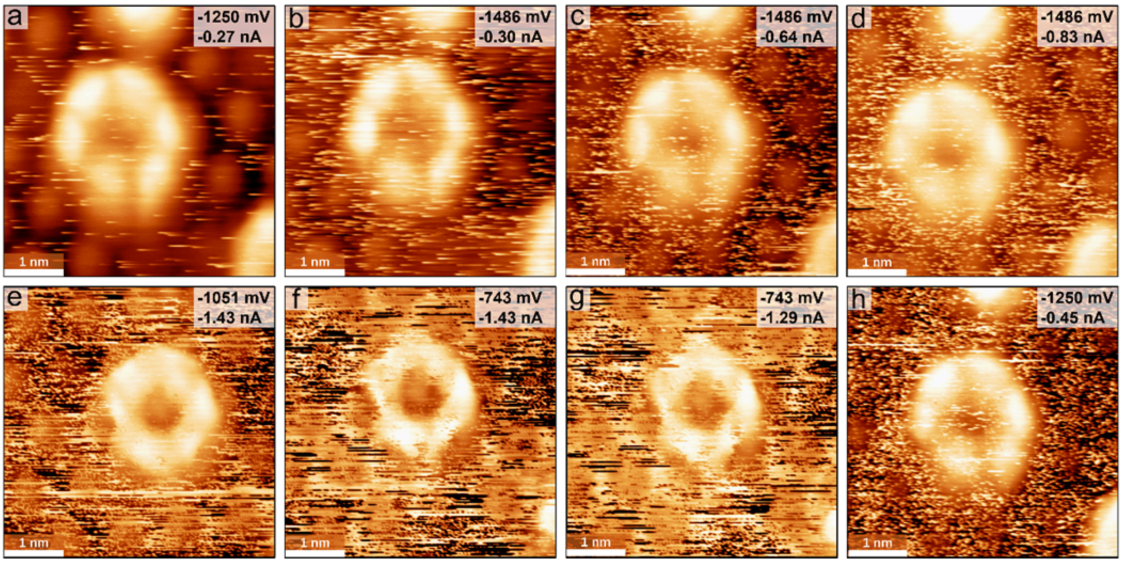

Supplementary Fig. 7 | a–h, STM images of a hexamer ring obtained by successive scanning with tunneling current varying from –0.27 nA to –1.43 nA and bias voltage ranging from –743 mV to –1486 mV. When bringing the tip close to the hexamer ring, its ring-like morphology is well maintained.

8. Covalent bonding between C_{60}-BCPMs

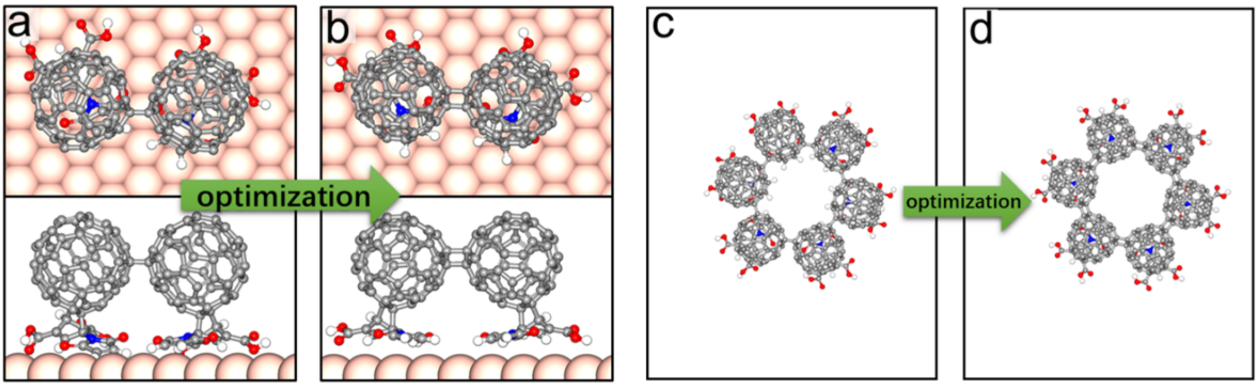

Supplementary Fig. 8| a, Initial structure, and **b,** optimized structure by DFT calculations for bonding between two C_{60}-BCPMs. When initially setting a single bond between them, the second bond forms after full relaxation. **c,** Initial structure, and **d,** optimized structure for bonding between the adjacent C_{60}-BCPMs in the HR. Similar to the case of two C_{60}-BCPMs, when we set a single bond between the adjacent C_{60}-BCPMs, the final structure after optimization shows two bonds between adjacent C_{60}-BCPMs.

9. Cycloaddition reactions between C_{60} and phenyl/maleimide ring of BCPM

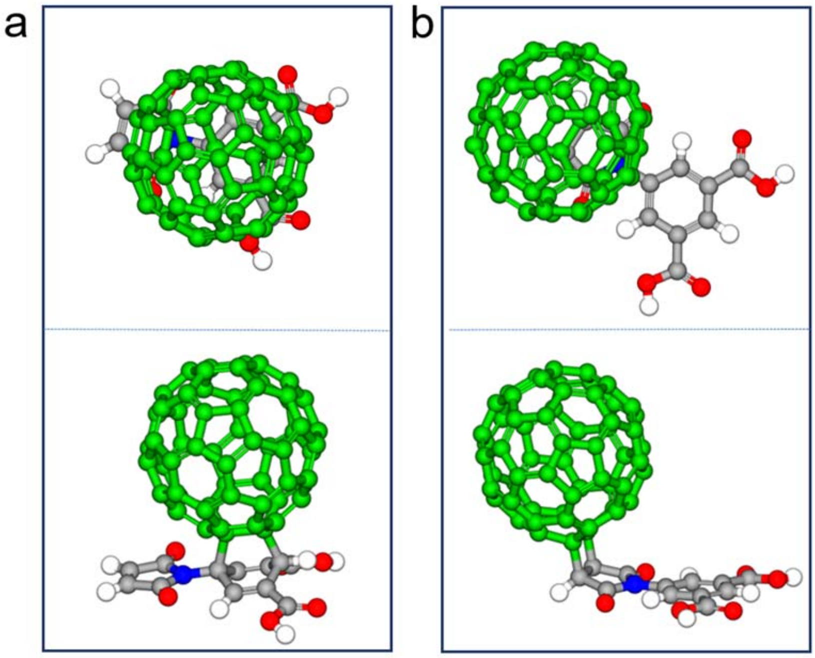

Supplementary Fig. 9 | Top and side views of DFT-optimized model of **a**, [4+2] cycloaddition between [6.6] bond of C_{60} and phenyl ring of BCPM, and **b,** [2+2] cycloaddition between [6,6] bond of C_{60} and C=C of BCPM's maleimide group.

10. Reaction pathway of interlayer cycloaddition between C_{60} and BCPM and lateral cycloaddition of C_{60}-BCPMs

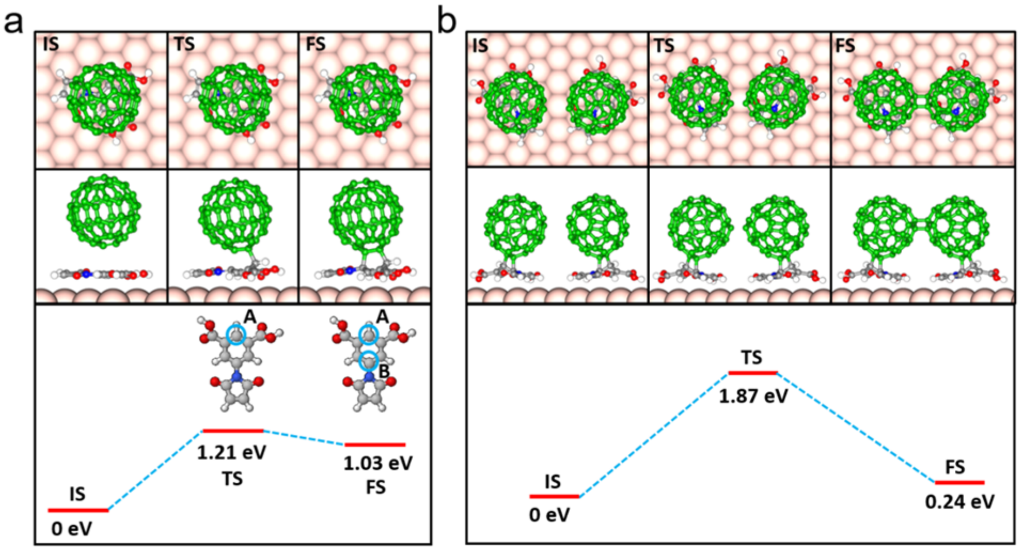

Supplementary Fig. 10 | Initial state (IS), transition state (TS) and final state (FS) geometries and the energy diagram of **a**, [4+2] cycloaddition between BCPM and C_{60} and **b,** [2+2] cycloaddition between two C_{60}-BCPMs, by the Cl-NEB calculations.

11. Influence of the functional groups of BCPM on its [4+2] cycloaddition with C_{60}

To explore the influence of the functional groups of BCPM on its [4+2] cycloaddition with C_{60}, we calculated [4+2] reactions of C_{60} with a number of different molecules in gas phase, including benzene (Supplementary Fig. 11a), benzene-1,3-dicarboxylic acid (i.e., benzene equipped with two carboxylic acid groups, Supplementary Fig. 11b), N-phenylmaleimide (i.e., benzene equipped with a maleimide group, Supplementary Fig. 11c), and compared them with cycloaddition of C_{60} and BCPM (Supplementary Fig. 11d). It is found that the equipped maleimide and carboxylic acid groups of BCPM can vary the reaction path of cycloaddition from two synchronous bonding between C_{60} and benzene into the nonsynchronous process where C_{60} bonds with Carbon A first and then with Carbon B. Moreover, the presence of maleimide and carboxylic acid groups lowers the energy barrier of [4+2] cycloaddition: the barrier for cycloaddition between C_{60} and benzene is 1.37 eV, while that between C_{60} and the phenyl ring of BCPM is 1.28 eV.

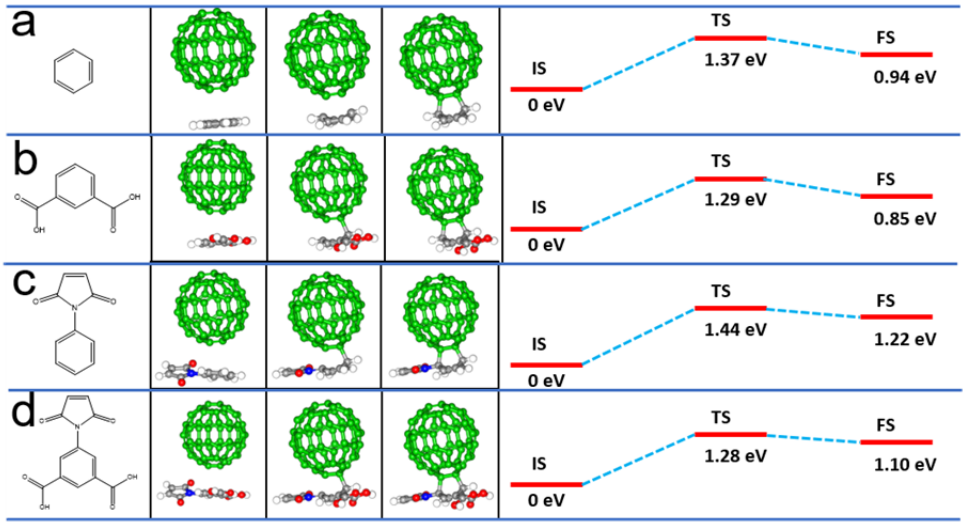

Supplementary Fig. 11 | The side views of IS, TS, FS structures and the energy diagrams for the [4+2] cycloaddition of C_{60} with **a,** benzene, **b,** benzene-1,3-dicarboxylic acid, **c,** N-phenylmaleimide, and **d,** BCPM in gas phase by the CI-NEB calculations.

12. Calculated reaction barrier for [2+2] cycloaddition between C_{60} parts of two C_{60}-BCPMs and between two pristine C_{60}s

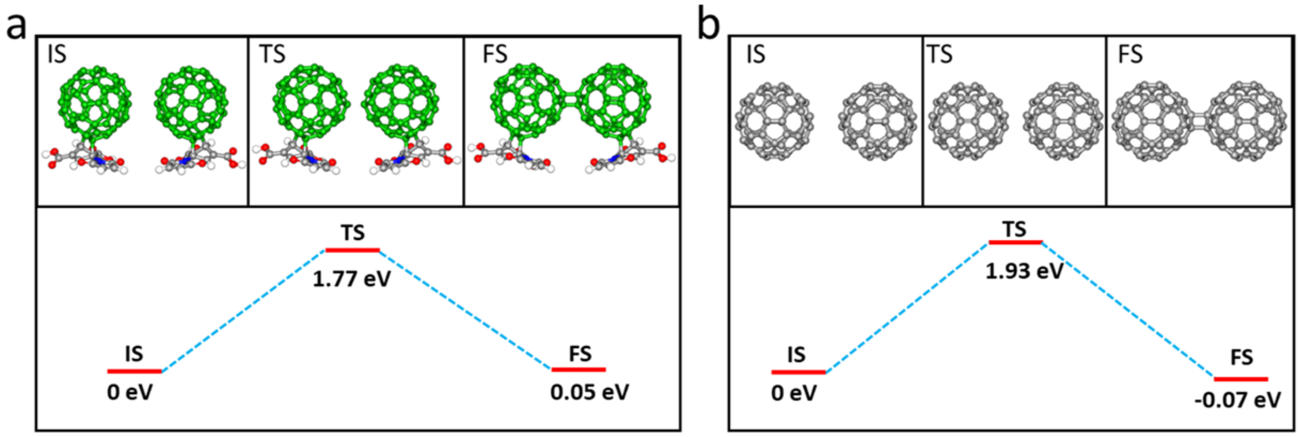

Supplementary Fig. 12 | Initial state (IS), transition state (TS) and final state (FS) geometry and the energy diagram for [2+2] cycloaddition between **a,** two C_{60}-BCPMs and **b,** two C_{60}s.

13. Formation of C_{60}-BCPM-C_{60} and BCPM-C_{60}-BCPM by interlayer cycloaddition

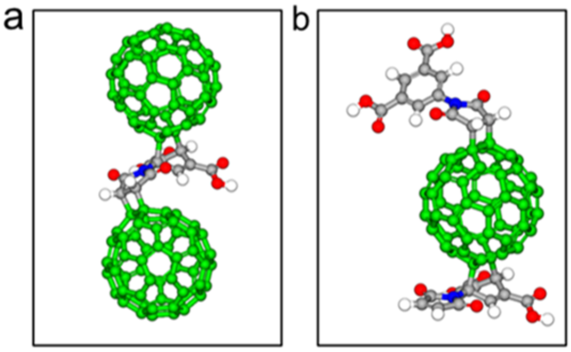

Supplementary Fig. 13 | The DFT-optimized structure of **a**, C_{60}-BCPM-C_{60} and **b**, BCPM-C_{60}-BCPM. As shown in panel a, BCPM can have cycloaddition with two C_{60} molecules: [4+2] cycloaddition between the phenyl ring of BCPM and one C_{60} molecule and [2+2] cycloaddition between the maleimide group of BCPM and another C_{60} on the other side of BCPM molecule's board. As shown in panel b, one C_{60} molecule can have cycloaddition with two BCPM molecules, one on the top and the other at the bottom.

Supplementary References

[1] Sarkar, S., Bekyarova, E. & Haddon, R.C. Chemistry at the Dirac Point: Diels–Alder Reactivity of Graphene. *Acc. Chem. Res*. **45**, 673–682 (2012).

[2] Zhu, S. E., Li, F. & Wang, G. W. Mechanochemistry of fullerenes and related materials. *Chem. Soc. Rev.* **42**, 7535–7570 (2013).

[3] Murata, Y., Kato, N., Fujiwara, K. & Komatsu, K. Solid-state [4+2] cycloaddition of fullerene C_{60} with condensed aromatics using a high-speed vibration milling technique. *J. Org. Chem.* **64**, 3483–3488 (1999).

[4] Fernández, I., Solà, M. & Bickelhaupt, F. M. Why do cycloaddition reactions involving C_{60} prefer [6,6] over [5,6] bonds? *Chem. Eur. J.* **19**, 7416–7422 (2013).

[5] Hamlin, T. A., Fernµndez, I. & Bickelhaupt, F. M. How dihalogens catalyze michael addition reactions. *Angew. Chem.*, *Int. Ed.* **58**, 8922–8926 (2019).
